# Inhibition of GCN2 Alleviates Cardiomyopathy in Type 2 Diabetic Mice via Attenuating Lipotoxicity and Oxidative Stress

**DOI:** 10.3390/antiox11071379

**Published:** 2022-07-16

**Authors:** Juntao Yuan, Fang Li, Bingqing Cui, Junling Gao, Zhuoran Yu, Zhongbing Lu

**Affiliations:** College of Life Science, University of Chinese Academy of Sciences, Beijing 100049, China; yuanjuntao@ucas.ac.cn (J.Y.); lif@ucas.ac.cn (F.L.); cuibingqing18@mails.ucas.ac.cn (B.C.); gaojunling17@mails.ucas.ac.cn (J.G.); yuzhuoran19@mails.ucas.ac.cn (Z.Y.)

**Keywords:** GCN2iB, diabetic cardiomyopathy, oxidative stress, lipid accumulation

## Abstract

Diabetic cardiomyopathy (DCM) is a kind of heart disease that affects diabetic patients and is one of the primary causes of death. We previously demonstrated that deletion of the general control nonderepressible 2 (GCN2) kinase ameliorates cardiac dysfunction in diabetic mice. The aim of this study was to investigate the protective effect of GCN2iB, a GCN2 inhibitor, in type 2 diabetic (T2D) mice induced by a high-fat diet (HFD) plus low-dose streptozotocin (STZ) treatments or deletion of the leptin receptor (db/db). GCN2iB (3 mg/kg/every other day) treatment for 6 weeks resulted in significant decreases in fasting blood glucose levels and body weight and increases in the left ventricular ejection fraction. GCN2iB treatment also attenuated myocardial fibrosis, lipid accumulation and oxidative stress in the hearts of T2D mice, which was associated with decreases in lipid metabolism-related genes and increases in antioxidative genes. Untargeted metabolomics and RNA sequencing analysis revealed that GCN2iB profoundly affected myocardial metabolomic profiles and gene expression profiles. In particular, GCN2iB increased myocardial phosphocreatine and taurine levels and upregulated genes involved in oxidative phosphorylation. In conclusion, the data provide evidence that GCN2iB effectively protects against cardiac dysfunction in T2D mice. Our findings suggest that GCN2iB might be a novel drug candidate for DCM therapy.

## 1. Introduction

Diabetic cardiomyopathy (DCM) is one of the major chronic complications associated with diabetes. The common symptoms of DCM include ventricular dilatation, cardiomyocyte enlargement, interstitial fibrosis and diastolic dysfunction [1,2]. Despite a significant rise in preclinical and clinical research over the last decade, the pathophysiology of DCM remains unknown, and there is no consensus on prevention or treatment strategies [3,4,5]. Considering that DCM profoundly affects the morbidity and mortality of diabetic patients, there is a need to find effective therapeutic approaches for DCM.

Although general control nonderepressible 2 (GCN2) is a well-known sensor of amino acid availability [6,7], it also plays an important role in other biological processes, including glucose and lipid metabolism [8,9,10], the immune response [11,12], memory formation [13], muscle atrophy [14] and cell apoptosis [15]. In the heart, GCN2 deficiency was found to attenuate transverse aortic constriction (TAC) and doxorubicin-induced cardiac dysfunction [16,17]. Interestingly, we also demonstrated that GCN2 deletion improves cardiac function in diabetic mice by reducing lipid accumulation, oxidative stress and cell death [18], suggesting that inhibition of GCN2 activity in the heart may be a potential approach for DCM therapy.

As an inhibitor of GCN2, GCN2iB was found to protect against cerebral I/R injury in mice by decreasing oxidative stress [19]. We recently showed that GCN2iB effectively decreased body weight, improved insulin sensitivity and attenuated hepatic steatosis and oxidative stress in obese mice [20]. As GCN2 is expressed at a lower level in the heart than in the liver [21], it is uncertain whether GCN2iB would exert beneficial effects in diabetic mice. In this study, we treated two type 2 diabetes mouse models with GCN2iB and examined the effects of GCN2iB on cardiac function, myocardial metabolism and oxidative stress.

## 2. Materials and Methods

### 2.1. Reagents and Antibodies

GCN2iB was purchased from MedChemExpress LLC (#HY-112654, Monmouth-Junction, NJ, USA). Streptozotocin (STZ) and dihydroethidium (DHE) were purchased from Sigma-Aldrich (#S0130 and #D7008, St. Louis, MO, USA). ELISA kits for 4-hydroxynonenal (4-HNE) and 3-nitrotyrosine (3′-NT) were obtained from Donggeboye Biological Technology Co. Ltd. (#DG30947M, Beijing, China) and Abcam PLC (#ab116691, Cambridge, UK), respectively. The triglyceride (TG) kit was obtained from Applygen Technologies Inc. (#E1013, Beijing, China). CF488A-conjugated wheat germ agglutinin (WGA) was obtained from Biotium Inc. (#29022, Fremont, CA, USA). The protease and phosphatase inhibitor cocktails were obtained from Roche (#04693124001, #4906837001, Basel, Switzerland). An antibody against atrial natriuretic peptide (ANP) was purchased from Wuhan Cloud-Clone Corp (#PAA225Bo01, Wuhan, China). Fatty acid synthase (FAS), glutathione peroxidase 4 (GPX4) and β-tubulin antibodies were purchased from Cell Signaling Technology (#3180, #59735, #2146, Danvers, MA, USA). Antibodies against peroxidase 2 (PRDX2) and thioredoxin 2 (Trx2) were obtained from Abcam (#ab109367, #ab185544). The Cidea antibody was purchased from Sino Biological Inc (#100879-T32, Beijing, China).

### 2.2. Animals and Treatment

Male C57BL/6J mice, leptin receptor deficient (db/db) mice and high-fat diet (HFD, 60% fat) were purchased from Beijing HFK Bioscience Co., LTD. (Beijing, China). As described previously, 8 weeks of HFD feeding and an intraperitoneal injection of STZ (formulated in 0.1 M citrate buffer, pH 4.5, 120 mg/kg) were used to induce type 2 diabetes [18]. Eight weeks after STZ injection, mice with hyperglycemia (6-h fasting blood glucose ≥ 11.1 mmol/L) were considered diabetic. The HFD plus STZ-induced diabetic mice or db/db mice were randomly divided into two groups (5 mice per group). Mice were treated with oil (used as a control) or GCN2iB (3 mg/kg) every other day via intraperitoneal injection for 6 weeks. At the end of the experiments, the mice were subjected to echocardiography and then euthanized using the spinal cord dislocation method.

During the whole experimental period, mice were maintained at 24 °C with a 12 h/12 h light/dark cycle and had free access to food and drinking water. Animal experiments were performed in accordance with the guidelines of the care and use of laboratory animals (Eighth edition, 2011).

### 2.3. Echocardiography

Transthoracic echocardiography was performed by a Vevo 2100 high-resolution imaging system equipped with a 30-MHz probe (MS400; VisualSonics, Toronto, ON, Canada), as previously described [18,22]. Mice were anesthetized with 0.8% isoflurane and placed on a heating pad during electrocardiogram recording.

### 2.4. Tissue Processing and Histopathology Staining

Mouse heart tissues were fixed with 4% paraformaldehyde and embedded in paraffin. Heart sections (5 μm) were stained with a Masson trichrome stain kit (#G1340, Solarbio Science & Technology Co. LTD, Beijing, China) to assess fibrosis. Frozen heart sections (4 μm) were stained with 10 μg/mL WGA or DHE for 30 min to assess myocyte cross-sectional area and superoxide levels, respectively. The following wavelength settings were used to obtain fluorescence images: WGA, excitation 485 nm/emission 530 nm; DHE, excitation 530 nm/emission 610 nm. Laser confocal microscope was used for image acquisition (Zeiss LSM 880, Germany). The relative fluorescence intensity was calculated by ImageJ (Version 1.50i, National Institutes of Health, Bethesda, MD, USA).

### 2.5. RNA Sequencing

RNA was extracted from heart tissue using TRIzol reagent (Invitrogen, MA, USA). After purification with DNase I and an rRNA Removal Kit (Human/Mouse/Rat) (Illumina, San Diego, CA, USA), a BGISEQ500 platform was used for library construction and RNA sequencing (BGI-Shenzhen, China). The detailed protocol/software for raw data reading, cleaning, mapping, gene expression level assessment, differentially expressed gene (DEG) screening and Kyoto Encyclopedia of Genes and Genomes (KEGG) enrichment analysis were described in our previous reports [23,24].

### 2.6. Untargeted Metabolomics Analysis

The detailed protocol for untargeted metabolomics analysis was described in our previous report [20]. Briefly, a mixture of 25 mg of heart tissue and 500 μL of extract solution (acetonitrile–methanol–water (2:2:1) with isotopically-labeled internal standard mixture) was homogenized and sonicated for 3 cycles and then kept at −40 °C for 1 h. After centrifugation at 12,000 rpm for 15 min at 4 °C, the supernatant was transferred to a fresh glass vial for subsequent LC-MS/MS analyses, which were performed on a UHPLC system (Vanquish, Thermo Fisher Scientific, Cambridge, MA) coupled to a Q Exactive HFX mass spectrometer (Orbitrap MS, Thermo Fisher Scientific, Cambridge, MA, USA). 

### 2.7. Western Blots

Proteins were extracted from heart homogenate with buffer (50 mM Tris-Cl, 150 mM NaCl, 100 μg/mL phenylmethylsulfonyl fluoride, protease and phosphatase inhibitor cocktail and 1% Triton X-100). After centrifugation at 12,000 g for 20 min at 4 °C, the supernatant was used for Western blot analysis. In brief, equal amounts of protein (10–40 μg) and molecular weight markers were loaded into the wells of SDS-PAGE gels. After running for 1–2 h at 100 V, the gels were transferred to PVDF membranes. Then, the membranes were blocked with blocking buffer (5% nonfat milk, 50 mmol/L Tris-HCl, 150 mmol/L NaCl and 0.1% Tween 20) for 1 h at room temperature. Next, the membranes were incubated with the appropriate dilutions (1:1000–1:2000) of primary antibody in blocking buffer overnight at 4 °C. After thorough washing, the membranes were incubated with the recommended dilution (1:5000–1:10,000) of conjugated secondary antibody in blocking buffer at room temperature for 1 h. After thorough washing and incubation with the chemiluminescent substrate, the membranes were placed into the ChemiDoc™ XRS+ Gel Imaging System (Bio-Rad Laboratories, Inc., Hercules, CA, USA) for visualization.

### 2.8. Real-Time PCR

A PrimeScript RT Reagent Kit (#RR036B, TaKaRa, Otsu, Japan) was used for cDNA synthesis, and a SYBR^®^ Premix Ex Taq™ II Kit was used for quantitative PCR (qPCR). The cycling conditions were as follows: initial denaturation at 95 °C for 30 s, followed by 40 cycles of 5 s at 95 °C, 30 s at 60 °C and 30 s at 72 °C. The results were normalized to 18S rRNA. The primers are listed in Table S1.

### 2.9. Statistical Analysis

Unpaired Student’s *t*-test or one-way ANOVA was used to analyze the data significant differences. All data are presented as the mean ± SD. GraphPad Prism software (Version 8, GraphPad Software Inc., CA, USA) was utilized for statistical analyses. A *p* < 0.05 was considered statistically significant.

## 3. Results

### 3.1. GCN2iB Attenuates Cardiac Dysfunction and Myocardial Fibrosis in HFD Plus STZ-Induced Diabetic Mice

**Figure 1:**
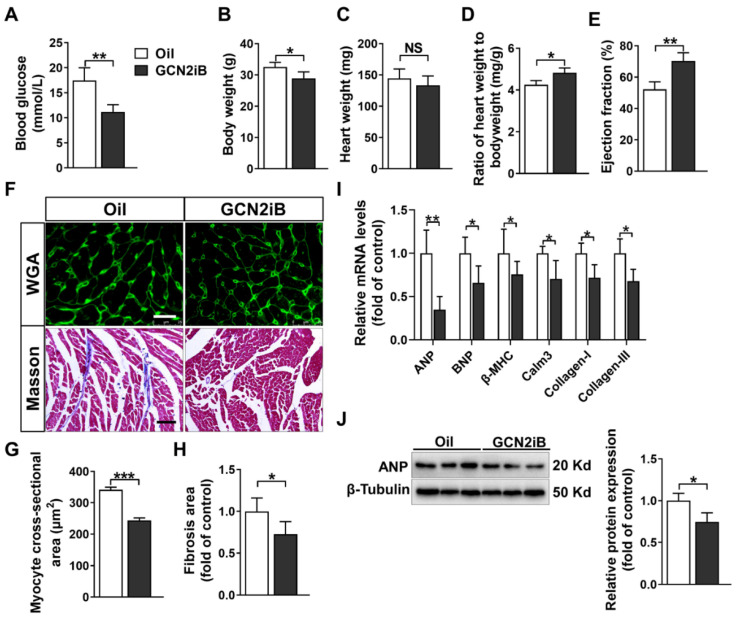
GCN2iB improves cardiac function and attenuates myocardial fibrosis in type 2 diabetic (T2D) mice. High-fat diet plus low-dose streptozotocin (STZ)-induced male type 2 diabetic mice were treated with oil or GCN2iB (3 mg/kg) every other day via intraperitoneal injection for 6 weeks. At the end of the experiments, fasting blood glucose levels (**A**), body weight (**B**), heart weight (**C**), the ratio of heart weight to body weight (**D**) and left ventricular ejection fraction (**E**) were measured. (**F**) Representative heart sections were stained with wheat germ agglutinin (WGA, upper panel, scale bar = 20 μm) and Masson trichrome stain (lower panel, scale bar = 100 μm). The myocyte cross-sectional area (**G**) and myocardial fibrosis (**H**) were quantified. (**I**) The mRNA levels of hypertrophic and fibrotic genes were measured by real-time qPCR. (**J**) Heart lysates were subjected to Western blotting to measure the expression of ANP. In Figure (**A**–**I**), N = 5; in Figure (**J**), N = 3; values are expressed as the means ± SD; * indicates *p* < 0.05; ** indicates *p* < 0.01; *** indicates *p* < 0.001.

After GCN2iB treatment, there were significant decreases in fasting blood glucose levels (11.18 ± 1.44 vs. 17.42 ± 2.57 mmol/L) and body weight (28.94 ± 2.05 vs. 32.54 ± 1.49 g) in type 2 diabetic mice (Figure 1A,B). GCN2iB treatment had no obvious effect on heart weight but increased the ratio of heart weight to body weight (Figure 1C,D). Echocardiographic examination revealed that GCN2iB treatment resulted in the significant improvement in cardiac systolic function, as evidenced by the increased left ventricular (LV) ejection fraction (EF) (70.41 ± 4.70 vs. 52.26 ± 4.14) (Figure 1E, Table S2). As demonstrated by Masson and WGA staining, GCN2iB decreased myocardial fibrosis and cardiac myocyte cross-sectional area in diabetic mice (Figure 1F,G). In addition, the mRNA levels of *ANP*, *BNP*, β-myosin heavy chain (*β-MHC*), *Calm3*, *Collagen-I* and *Collagen-III* were significantly decreased in the hearts of GCN2iB-treated diabetic mice (Figure 1I). The GCN2iB-induced downregulation of myocardial ANP was further confirmed by Western blot (Figure 1J). 

### 3.2. GCN2iB Affects Myocardial Metabolomic Profiles in HFD Plus STZ-Induced Diabetic Mice

**Figure 2:**
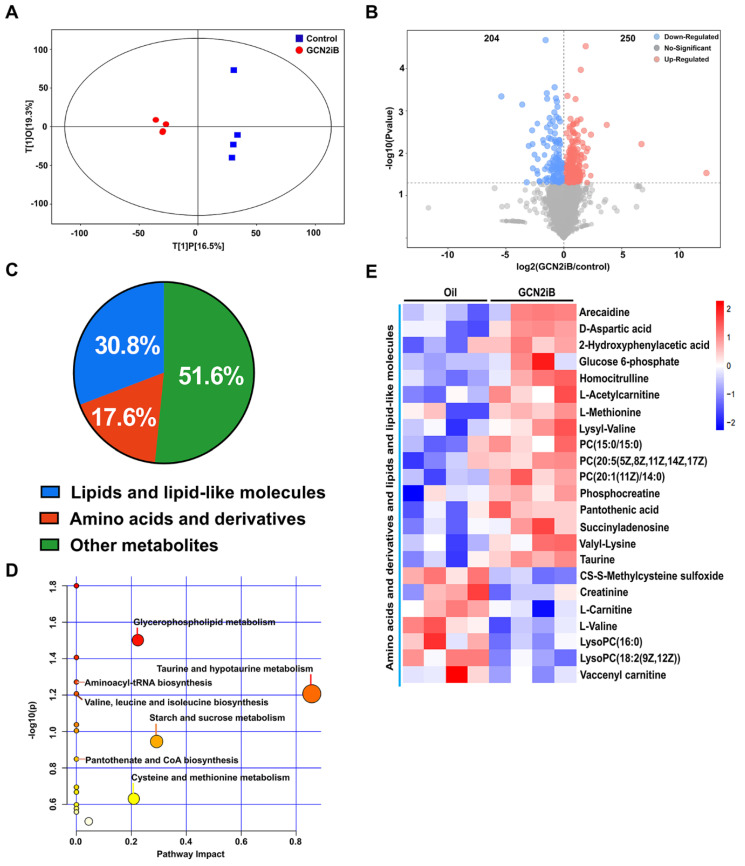
Effect of GCN2iB treatment on myocardial metabolomic profiles in T2D mice. (**A**) Orthogonal projections to latent structures discriminant analysis (OPLS-DA) models for the oil- and GCN2iB-treated groups are presented as score scatter plots. (**B**) The changes in myocardial metabolites in T2D mice are presented in a volcano plot. (**C**) A pie chart is used to illustrate the percentages of major metabolites of T2D mice. (**D**) KEGG pathway enrichment analysis of significantly different metabolites. (**E**) The significantly changed amino acid- and lipid-related metabolites caused by GCN2iB treatment are presented as a heatmap.

It is well known that hyperglycemia profoundly affects cardiac substrate metabolism, which contributes greatly to the pathogenesis of DCM [25,26]. To investigate the effect of GCN2iB on cardiac metabolism, an untargeted metabolomics approach was applied to determine the changes in myocardial metabolites in diabetic hearts. The orthogonal projection of the latent structure discriminant analysis (OPLS-DA) score plot revealed that primary metabolic components were different between the oil- and GCN2iB-treated hearts (Figure 2A). Based on the selection criteria of variable importance in the projection (VIP) > 1.0 and Student’s *t*-test *p* value < 0.05, the fold changes of 454 differential metabolites (250 increased and 204 decreased) were visualized in a volcano plot (Figure 2B). Subsequently, the differential metabolites were classified according to their categories: lipids and lipid-like molecules (30.8%); amino acids and derivatives (17.6%); and others (51.6%) (Figure 2C). Forty-eight differential metabolites were screened out with the selection criteria of VIP > 1.0 and false discovery rate (FDR)-adjusted p-value < 0.05. Then, we used MetaboAnalyst software (version 5.0, http://www.metaboanalyst.ca/ (accessed on 15 April 2022)) to map those metabolites to the appropriate physiological pathways. As revealed by the KEGG pathway enrichment analysis, the perturbed metabolic pathways in GCN2iB-treated diabetic hearts were related to the following functions: glycerophospholipid metabolism; valine, leucine and isoleucine biosynthesis; taurine and hypotaurine metabolism; aminoacyl-tRNA biosynthesis; starch and sucrose metabolism; pantothenate and CoA biosynthesis; and cysteine and methionine metabolism (Figure 2D). The relative levels of representative metabolites involved in amino acid metabolism as well as lipid and lipid-like metabolism are presented in a heatmap (Figure 2E), in which the levels of arecaidine, D-aspartic acid, 2-fydroxyphenylacetic acid, glucose 6-phosphate, homocitrulline, L-acetylcarnitine, L-methionine, lysyl-valine, PC(15:0/15:0), PC(20:5(5Z,8Z,11Z,14Z,17Z), PC(20:1(11Z)/14:0), phosphocreatine, pantothenic acid, succinyladenosine, valyl-lysine and taurine were increased while the levels of CS-S-methylcysteine sulfoxide, creatinine, L-carnitine, L-valine, lysoPC(16:0), lysoPC(18:2(9Z,12Z)) and vaccenyl carnitine were decreased in the hearts of GCN2iB-treated diabetic mice.

### 3.3. GCN2iB Affects Gene Expression Profiles in T2D Mice

**Figure 3:**
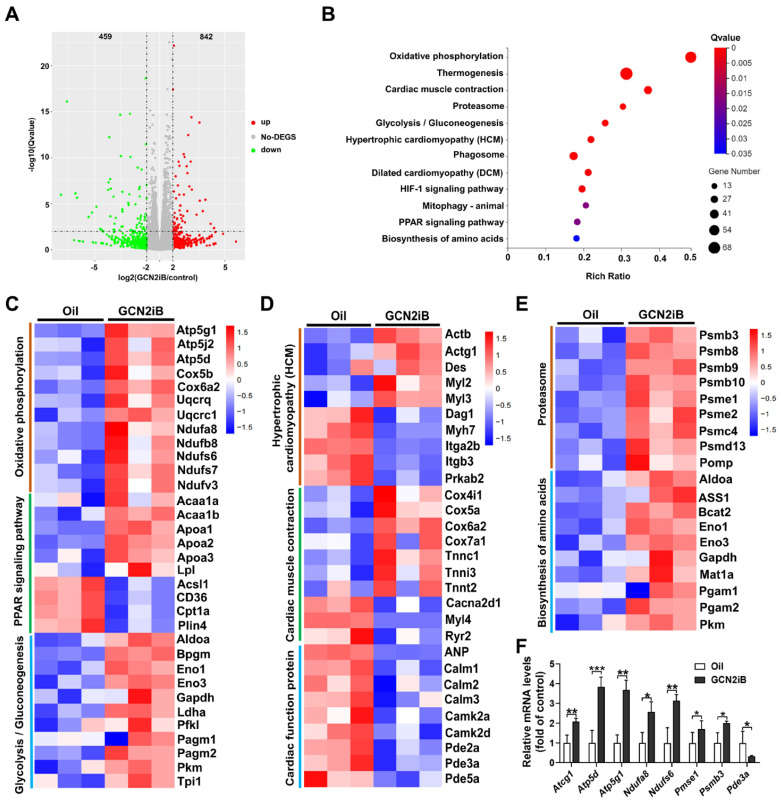
GCN2iB affects gene expression profiles in T2D mice. (**A**) A volcano plot was used to show the fold changes in differentially expressed genes (DEGs) in the control group vs. the GCN2iB group. (**B**) Twelve significantly enriched KEGG pathways are listed as an advanced bubble chart. (**C**) The gene expression profiles of the DEGs that were involved in oxidative phosphorylation, the PPAR signaling pathway and glycolysis/gluconeogenesis pathways are shown in the heatmap. (**D**,**E**) The gene expression profiles of the DEGs that were involved in cardiac function, proteasome and biosynthesis of amino acids pathways are shown in the heatmap. (**F**) The mRNA levels of some randomly selected differentially expressed genes were measured by qPCR. In Figure (**F**), N = 5, values are expressed as the means ± SD; * indicates *p* < 0.05; ** indicates *p* < 0.01; *** indicates *p* < 0.001.

To elucidate the molecular protective mechanism of GCN2iB, we performed RNA sequencing to determine the effect of GCN2iB on global changes in the gene expression profile in diabetic hearts. We identified 1301 DEGs (842 upregulated and 459 downregulated) in the oil group vs. the GCN2iB group. The fold changes in DEGs are displayed in the volcano plot (Figure 3A). KEGG pathway enrichment analysis revealed that these DEGs were significantly enriched in metabolism- and cardiomyopathy-related pathways, such as oxidative phosphorylation, thermogenesis, cardiac muscle contraction, proteasome, glycolysis/gluconeogenesis, hypertrophic cardiomyopathy (HCM), phagosome, dilated cardiomyopathy, HIF-1 signaling pathway, mitophagy—animal, PPAR signaling pathway and biosynthesis of amino acids (Figure 3B). In oxidative phosphorylation-related DEGs, the expression levels of *Atp5g1*, *Atp5j2*, *Atp5d*, *Cox5b*, *Cox6a2*, *Uqcrq*, *Uqcrc1*, *Ndufa8*, *Ndufb8*, *Ndufs6/7*, *Ndufs7* and *Ndufv7* were increased in the GCN2iB group. In PPAR signaling pathway-related DEGs, GCN2iB increased the expression of acetyl-coenzyme A acyltransferase 1 (*Acaa1a/b*), apolipoprotein A1/A2/A3 (*Apoa1/2/3*) and lipoprotein lipase (*Lpl*) but decreased the expression of acyl-CoA synthetase long chain family member 1 (*Acsl1*), *CD36*, carnitine palmitoyltransferase 1A (*Cpt1a*) and perilipin 4 (*Plin4*). In the glycolysis/gluconeogenesis pathway, the levels of *Aldoa*, *Bpgm*, *Eno1/3*, *Gapdh*, *Ldha*, *Pfkl*, *Pagm1/2*, *Pkm* and *Tpi1* were increased in the GCN2iB group (Figure 3C). In HCM and cardiac muscle contraction pathways-related DEGs, the expression levels of *Actb*, *Actg1*, *Des*, *Myl2/3*, *Cox4i1*, *Cox5a*, *Cox6a2*, *Cox7a1* and *Tnnc1/i3/t2* were increased, while the expression levels of the genes (*Dag1*, *Myh7*, *Itga2b*, *Itgb3*, *Prkab2*, *Ryr2*, *Cacna2d1* and *Myl4*) that promote cardiomyopathy were decreased in the GCN2iB group. Among the DEGs involved in the dilated cardiomyopathy pathway, the expression levels of *ANP*, *Calm1/2/3*, *Camk2a/d* and *Pde2/3/5a* were decreased in the GCN2iB group (Figure 3D). In addition, GCN2iB treatment also increased the expression of a series of genes involved in the proteasome pathway (e.g., *Psmb3/8/9/10*, *Psme1/2*, *Psmc4*, *Psmd13* and *Pomp*) and the biosynthesis of amino acids pathway (e.g., *Aldoa*, *ASS1*, *Bcat2*, *Eno1/3*, *Gapdh*, *Mat1a*, *Pgam1/3* and *Pkm*) metabolism (Figure 3E). To validate the RNA sequencing results, we measured the mRNA levels of some randomly selected genes. As revealed by the qPCR results, GCN2iB significantly increased the expression of *Atcg1*, *atp5d*, *atp5j1*, *Ndufa8*, *Ndufs6*, *Psmb3* and *Psme1* but decreased the expression of Pde3a in diabetic hearts (Figure 3F).

### 3.4. GCN2iB Alleviates Myocardial Lipid Accumulation and Oxidative Stress in T2D Mice

**Figure 4:**
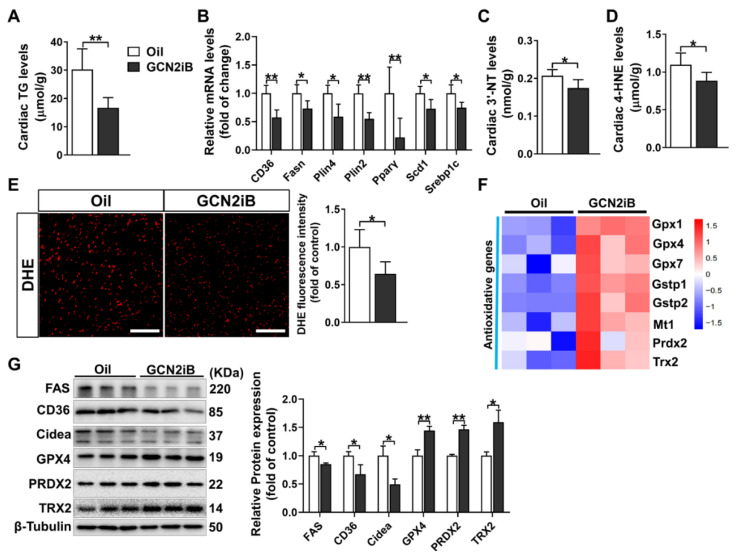
GCN2iB ameliorates myocardial lipid accumulation and oxidative stress in T2D mice. (**A**) Cardiac triglyceride (TG) levels were measured in oil- and GCN2iB-treated T2D mice. (**B**) The mRNA levels of lipid metabolism-related genes were measured. (**C**,**D**) Cardiac 3′-nitrotyrosine (3′-NT) and 4-hydroxynonenal (4-HNE) levels were measured. (**E**) Representative heart sections were stained with dihydroethidium (DHE), and the relative fluorescence intensity was quantified. Scale bar = 50 μm. (**F**) The expression profiles of some antioxidative genes are displayed as a heatmap. (**G**) Heart lysates were examined by Western blot. In Figure (**A**–**E**), N = 5; in Figure 4 (**F**,**G**), N = 3; values are expressed as the means ± SD; * indicates *p* < 0.05; ** indicates *p* < 0.01.

Myocardial lipid accumulation is a hallmark of DCM [27]. As shown in Figure 4A, GCN2iB treatment significantly decreased myocardial TG in T2D mice. In addition, GCN2iB also decreased the mRNA levels of lipid metabolism-related genes, including *CD36*, *Fasn*, *Plin4*, *Plin2*, *Pparγ*, *SCD1* and *Srebp1c* (Figure 4B). Lipid accumulation is associated with oxidative stress, which plays an important role in the development of DCM [28]. GCN2iB treatment resulted in significant decreases in myocardial 3′-NT and 4-HNE levels (Figure 4C,D). GCN2iB also decreased superoxide levels in diabetic hearts (Figure 4E). To investigate the underlying mechanism for the decreased oxidative stress in GCN2iB-treated hearts, some antioxidative gene expression profiles are displayed as a heatmap (Figure 4F). We found that glutathione peroxidase (*Gpx1/4/7*), glutathione S-transferase Pi (*Gstp1/2*), metallothionein 1 (*Mt1*), peroxiredoxin 2 (*Prdx2*) and thioredoxin 2 (*Trx2*) were significantly upregulated in the hearts of GCN2iB-treated diabetic mice. The upregulation of GPRX4, PRDX2 and TRX2 was further confirmed by Western blotting. We also found that GCN2iB significantly decreased the protein expression levels of FAS and CIDEA (Figure 4G). Together, these results suggest that GCN2iB may protect against DCM by attenuating lipid accumulation and oxidative stress.

### 3.5. GCN2iB Improves Cardiac Function and Alleviates Myocardial Lipid Accumulation and Oxidative Stress in db/db Mice

**Figure 5:**
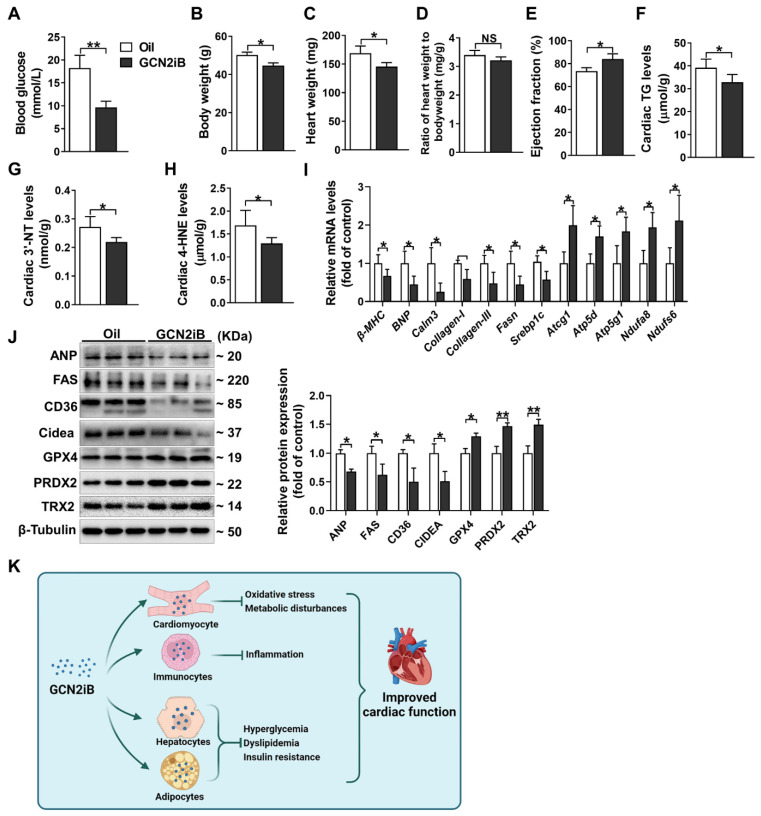
GCN2iB improves cardiac function and alleviates myocardial oxidative stress and lipid accumulation in db/db mice. Db/db mice were treated with GCN2iB (3 mg/kg) every other day via intraperitoneal injection for 6 weeks. Then, fasting blood glucose levels (**A**), body weight (**B**), heart weight (**C**), ratio of heart weight to body weight (**D**), left EF (**E**), cardiac TG levels (**F**), 3′-NT levels (**G**) and 4-HNE levels (**H**) were measured. (**I**) The mRNA levels of genes related to hypertrophy, fibrosis, lipid metabolism and oxidative phosphorylation were measured. (**J**) Heart lysates were examined by Western blot. (**K**) Schematic diagram shows the potential target cell affected by GCN2iB in T2D mice. In Figure (**A**–**I**), N = 5; in Figure (**J**), N=3; values are expressed as the means ± SD; * indicates *p* < 0.05; ** indicates *p* < 0.01.

To determine the generalizability of GCN2iB in improving DCM, we also treated db/db mice with GCN2iB via intraperitoneal injections. GCN2iB decreased blood glucose levels (9.64 ± 1.38 vs. 18.26 ± 2.78 mmol/L) (Figure 5A), body weight (44.67 ± 1.38 vs. 50.16 ± 1.57 g) (Figure 5B) and heart weight (145.7 ± 6.9 vs. 169.1 ± 12.5 mg) (Figure 5C), but had no obvious effect on the heart weight to body weight ratio (Figure 5D). Echocardiography showed that GCN2iB-treated mice exhibited higher LV EF values than control mice (84.05 ± 4.99 vs. 73.43 ± 2.99) (Figure 5E). GCN2iB treatment resulted in significant decreases in myocardial TG, 3′-NT and 4-HNE levels in db/db mice (Figure 5F–H). qPCR results showed that GCN2iB decreased the mRNA levels of *β-MHC*, *BNP*, *Calm3*, *Collagen-I*, *Collagen-III*, *Fasn* and *Srebp1c* but increased the levels of *Atcg1*, *Atp5d*, *Atp5j1*, *Ndufa8* and *Ndufs6* (Figure 5I). Western blotting revealed that GCN2iB treatment significantly decreased the protein expression of ANP, FAS, CD36 and Cidea and increased the expression of GPX4, PRDX2 and TRX2 in the hearts of db/db mice (Figure 5J).

## 4. Discussion

The major findings of the present study are that GCN2iB improved cardiac systolic function in two T2D mouse models, which was associated with reduced blood glucose, fibrosis, lipid accumulation and oxidative stress. Moreover, the effects of GCN2iB on the gene expression profile and myocardial metabolites were also determined.

There is no doubt that chronic hyperglycemia is the most important mechanism for the development of DCM [25], which induces the formation of advanced glycation end products (AGEs) through nonenzymatic glycation of proteins, lipids and lipoproteins [29]. We previously showed that GCN2 deficiency decreased blood glucose levels in both HFD plus STZ-induced T2D mice and HFD-induced obese mice [10,18]. We also demonstrated that GCN2iB decreased blood glucose levels in obese mice [20]. Thus, the finding that GCN2iB decreased fasting blood glucose levels in T2D mice was fully anticipated. Considering that GCN2 expression in the liver is high and that the liver plays an essential role in controlling systemic glucose homeostasis, we speculated that the antihyperglycemic effect of GCN2iB is associated with the inhibition of hepatic GCN2.

The present study showed that GCN2iB decreased cardiac TG levels in T2D mice, suggesting that GCN2iB might protect against DCM by attenuating lipid accumulation. In the diabetic heart, CD36 is upregulated and contributes to lipid accumulation by promoting the uptake of fatty acids [30]. As CD36 deficiency exerts beneficial effects in lipid-mediated cardiac dysfunction models [31,32], it is believed that CD36 is a detrimental factor for DCM. We previously showed that GCN2 deficiency repressed the upregulation of CD36 and attenuated lipotoxicity in diabetic hearts [18]. The lipid droplet proteins Plin2/4 are important for lipid storage in the heart. Cardiac overexpression of Plin2 induces atrial steatosis in aged mice [33], while inactive Plin4 reduces cardiac lipid accumulation in mice [34]. Therefore, our finding that GCN2iB decreased the expression of CD36 and other lipid metabolism-related genes suggests a potential mechanism for the GCN2iB-mediated reduction in cardiac TG levels in T2D mice.

The finding that GCN2iB decreased myocardial 3′-NT and 4-HNE levels suggested that the protective mechanism of GCN2iB is partially associated with repressing oxidative stress. We previously showed that GCN2 deficiency attenuated pressure overload-, doxorubicin- and diabetes-induced myocardial oxidative stress in mice [16,17,18]. Moreover, GCN2iB was found to attenuate cerebral ischemia/reperfusion injury in mice through the blockage of FoxO3a-regulated ROS production [19]. GCN2iB also alleviates hepatic oxidative stress in obese mice by activating the Nrf2 signaling pathway [20]. Thus, it is anticipated that GCN2iB functions as an antioxidant to protect against DCM. The finding that *Gpx1/4/7*, *Gstp1/2*, *Mt1*, *Prdx2* and *Trx2* were upregulated in GCN2iB-treated hearts suggested that the antioxidative property of GCN2iB might be associated with the upregulation of antioxidative genes. Mitochondrial respiration impairment is an important source for ROS in the development of DCM [35]. Phosphocreatine was found to improve oxidative phosphorylation and electron transport capacity in the mitochondria of diabetic hearts [36]. The findings that GCN2iB increased myocardial phosphocreatine content and the expression of genes involved in oxidative phosphorylation suggested that GCN2iB might decrease mitochondria-mediated ROS production by normalizing mitochondrial respiratory function. Moreover, it has been reported that taurine improves cardiac function and attenuates myocardial oxidative stress in type 1 diabetic rats [37]. Therefore, the protective effect of GCN2iB may also be related to the regulation of endogenous metabolites.

The genetic knockout approach is useful and reliable for studying specific gene function. With *Gcn2*^−/−^ mice, we previously demonstrated that GCN2 deletion improved cardiac function in diabetic mice [18]. Here, we further confirmed that GCN2iB, a GCN2 specific inhibitor, exerted similar cardioprotective effects in T2D mice, indicating that other GCN2-specific inhibitors, including SP600125 [38] and GCN2-in-1 (A92) [39], may also have beneficial effects in DCM therapy. Since the bioactive effect of GCN2iB has been observed in multiple cells or organs [19,20,40,41], the cardioprotective effect of GCN2iB seems to result from both systemic and cardiac tissue action. As illustrated in Figure 5k, we proposed that the target cell affected by GCN2iB includes cardiomyocyte, hepatocyte, adipocyte and immune cells.

## 5. Conclusions

In summary, our study indicates that GCN2iB improves cardiac function in T2D mice by attenuating hyperglycemia, myocardial lipid accumulation and oxidative stress. Our results suggest that GCN2iB administration is a potential strategy for DCM therapy.

## Data Availability

The data presented in this study are available on reasonable request from the corresponding author. The sequencing data for clean reads generated by this study have been deposited in the NCBI Sequence Read Archive (SRA) database (accession number: PRJNA846613).

## Supplementary Materials

The following supporting information can be downloaded at https://www.mdpi.com/article/10.3390/antiox11071379/s1, Table S1: The quantitative real-time PCR primer information; Table S2: Cardiac function data for oil- and GCN2iB-treated type 2 diabetic mice. Table S2: Cardiac function data for oil- and GCN2iB-treated db/db mice.
