# Supplementary material for: Inhibition of GCN2 Alleviates Cardiomyopathy in Type 2 Diabetic Mice via Attenuating Lipotoxicity and Oxidative Stress
Source: Antioxidants (Basel). 2022 Jul 16;11(7):1379. doi: 10.3390/antiox11071379 (PMC9312289; doi:10.3390/antiox11071379)
Supplement: Supplementary file 1 [file antioxidants-11-01379-s001.zip › antioxidants-1787700-Supplementary.pdf]

Supplemental Files

Inhibition of GCN2 alleviates cardiomyopathy in type 2 diabetic mice via attenuating lipotoxicity and oxidative stress

Juntao Yuan[#], Fang Li[#], Bingqing Cui, Junling Gao, Zhuoran Yu, Zhongbing Lu[*]

College of Life Science, University of Chinese Academy of Sciences, Beijing, 100049, China

Running Title: GCN2 inhibitor improves diabetic cardiomyopathy

# These authors contributed equally to this work.

* Correspondence

Zhongbing Lu, PhD

E-mail: luzhongbing@ucas.ac.cn

College of Life Science, University of Chinese Academy of Sciences

19A Yuquanlu, Beijing, 100049, China

Fax: 86-10-69672630; Tel: 86-10-69672630

Table S1. The quantitative real-time PCR primer information.

Genes name	Accession	Primers	Sequences (5'-3')	Length (bp)
Anp	NM_008725.3	Forward	5'- GGGGGTAGGATTGACAGGAT -3'	99
		Reverse	5'- CAGAATCGACTGCCTTTTCC -3'	
CD36	NM_001159555.1	Forward	5'-CCTGCAAATGTCAGAGGAAA-3'	92
		Reverse	5'-GCGACATGATTAATGGCACA-3'	
Ndufa8	NM_026703.3	Forward	5'- GCGGAGCCTTTCACAGAGTA-3'	228
		Reverse	5'- AATCACAGGGTTGGGCTCTG-3'	
Cidea	NM_007702.2	Forward	5'- AGAAGGTCCTACTGACCCCC -3'	266
		Reverse	5'- ACCCGGTGTCCATTTCTGTC -3'	
Ndufs6	NM_010888.2	Forward	5'- TCGGGGTTCAAGTGTCGC-3'	211
		Reverse	5'- TTATGTACACCTTGGGGTGGC-3'	
ATP5d	NM_001347092.1	Forward	5'- AGTCAGAACTGTCAGGTGCG -3'	376
		Reverse	5'- ACAGAACACTCAGTTGGTTCCT-3'	
Fasn	NM_007988.3	Forward	5'- CTCCACAGCTCTTCCAGTGAG -3'	246
		Reverse	5'- TCTCTAGAGGGCTTGCACCA -3'	
Bnp	NM_001287348.2	Forward	5'-CTGCTGGAGCTGATAAGAGA-3'	176
		Reverse	5'-TGCCCAAAGCAGCTTGAGAT-3'0	
β-Mhc	NM_001361607.1	Forward	5'- GCTGCCCCATATATACAGCCC -3'	265
		Reverse	5'- GGAGCCACCTTGGAACACTT -3'	
Srebp1c	NM_001313979.1	Forward	5'- CTGTCTCACCCCCAGCATAG -3'	119
		Reverse	5'- GATGTGCGAACTGGACACAG -3'	
Plin2	NM_001403711.1	Forward	5'- CTCGTCCCTCAGCTCTCCT -3'	347
		Reverse	5'- TTGGCCACTCTCATCACCAC -3'	
Plin4	NM_001372234.1	Forward	5'- TCTGAACAGACAGCTGGAGA-3'	120
		Reverse	5'- CAGTCCACCCTGGACCATTC-3'	
Actg1	NM_001313923.1	Forward	5'- TCGAACACGGCATTGTCACT-3'	163
		Reverse	5'- GGAACAGAACCCTGCGTCAT-3'	
Pparγ	NM_001127330.2	Forward	5'- GCGGAAGAAGAGACCTGGG -3'	116

		Reverse	5'- GTGTGACTTCTCCTCAGCCC -3'	
Scd1	NM_009127.4	Forward	5'- CGCTGGCACATCAACTTCAC-3'	162
		Reverse	5'- AGGAACTCAGAAGCCCAAAGC -3'	
ATP5g1	NM_001161419.1	Forward	5'-GGAGTGGGAGTGCAGATTGA-3'	296
		Reverse	5'-ACCAAACACTGTGCCAATGC-3'	
Collagen I	NM_007742.4	Forward	5'- CACCCCAATCTGGTTCCCTC -3'	333
		Reverse	5'- CATAAGCCAAGTGGGCAGGA -3'	
Collagen III	NM_009930.2	Forward	5'- GAGGAATGGGTGGCTATCCG-3'	316
		Reverse	5'- TTGCGTCCATCAAAGCCTCT-3'	
Pde3a	NM_018779.2	Forward	5'-TCTGCAAGGCTGAAACTTCG-3'	248
		Reverse	5'-CTCTCCAACAACAGCCGGAG-3'	
Psme1	NM_011189.1	Forward	5'- TGGTCACTACCTGGTTGCAG-3'	140
		Reverse	5'- CTTGGAGATCTGCGTGTGGA-3'	
Psmb3	NM_011971.4	Forward	5'- CTTCCACTCCAGCGCAATCA-3'	112
		Reverse	5'- CTGGATCCCGAAACGTCTG-3'	
18S	NR_003278.3	Forward	5'-AGGAATTGACGGAAGGGCACCAC-3'	327
		Reverse	5'-GTGCAGCCCCGGACATCTAAGG-3'	

Table S2. Cardiac function data for Oil and GCN2iB-treated type 2 diabetic mice.

Parameter	Oil (Control)	GCN2iB (Treatment)
LVAW;d (mm)	0.85±0.06	0.95±0.05*
LVAW;s (mm)	1.16±0.05	1.49±0.09*
LVID;d (mm)	4.0±0.31	3.68±0.27
LVID;s (mm)	2.95±0.21	2.39±0.36*
LVPW;d (mm)	1.0±0.09	0.94±0.13
LVPW;s (mm)	1.17±0.03	1.41±0.12*
EF (%)	52.26±4.14	70.41±4.70**
FS (%)	26.46±2.66	39.14±3.88**

LVAW;d: Left ventricular end-diastolic anterior wall thickness, LVAW;s: Left ventricular end-systolic anterior wall thickness; LVID;d: Left ventricular cavity diastolic dimension; LVID;s: left ventricular end-systolic internal diameter; LVPW;d: Left ventricular end-diastolic posterior wall thickness; LVAW;s: Left ventricular end-systolic anterior wall thickness; EF: Ejection fraction; FS: fractional shortening; N=5, Data are mean ± SD. * indicates $p<0.05$, ** indicates $p<0.01$.

Table S3. Cardiac function data for Oil and GCN2iB-treated db/db mice.

Parameter	Oil (Control)	GCN2iB (Treatment)
LVAW;d (mm)	0.90±0.04	1.06 ±0.03*
LVAW;s (mm)	1.40±0.15	1.55±0.13
LVID;d (mm)	3.92±0.14	3.57±0.09*
LVID;s (mm)	2.95±0.13	1.70±0.20*
LVPW;d (mm)	0.98±0.08	0.99±0.10
LVPW;s (mm)	1.51±0.07	1.74±0.10*
EF (%)	73.43±2.99	84.05±4.99*
FS (%)	41.91±2.25	52.32±5.62*

N=5, Data are mean ± SD. * indicates $p<0.05$, ** indicates $p<0.01$.
